# An Examination of Supplementary Homicide Reports and Fatal Encounters Data on Police-Caused Homicides

**DOI:** 10.7759/cureus.99376

**Published:** 2025-12-16

**Authors:** Jonathan Dirlam

**Affiliations:** 1 Sociology, University at Albany, State University of New York (SUNY), Albany, USA

**Keywords:** fatal encounters, multiple imputation, police-caused homicide, police lethal force, shr

## Abstract

The two official sources of police-caused homicides, National Vital Statistics System and FBI Supplementary Homicide Reports, have been widely criticized for underreporting and measurement error. Independent data collections emerged in the 2010s after several high-profile incidents of the police killing unarmed citizens. These collections include Fatal Encounters (FE), Mapping Police Violence, The Guardian, and The Washington Post. The validity of these datasets is unknown, and none of them extend before the year 2000. Researchers interested in studying police-caused homicides before 2000 are left with the two official sources with known data problems. This study performs multiple imputation on Supplementary Homicide Reports data to obtain national estimates from 1980 to 2010. We present a comparison and validity check with FE to our imputed estimates and find FE data to be underreported in the early 2000s compared to the early 2010s. We also perform a statistical analysis from 1980 to 2010 on police-caused homicides using non-imputed and imputed values and compare results.

## Introduction

Any analysis on police-caused homicides (PCH), where police have killed a citizen, that extends before 2000 must rely on one of the two official government sources: FBI Supplementary Homicide Reports (SHR) and National Vital Statistics System (NVSS) [1-3]. NVSS is based on death certificate information and suffers from measurement error as police involvement is not always specified by the coroner [3]. SHR is based on voluntary participation from law enforcement agencies that submit these reports to the FBI every month when they have at least one homicide. SHR suffers from missing data as not all agencies submit all of their monthly reports [1]. This dataset also relies on self-reports from police who may be incentivized to either underreport incidents or inaccurately describe the circumstances surrounding them to benefit the police. The data flaws in the two official sources of police-caused homicides help explain why The Washington Post found almost twice as many in 2015 compared to the SHR average in the prior decade [4].

After the murders of Michael Brown and Eric Garner in 2014, several independent data collections on PCH emerged, including (i) The Guardian from 2015-2016, (ii) The Washington Post from 2015 to 2024 before they stopped as of January 1, 2025, (iii) Mapping Police Violence from 2013 to present, and (iv) Fatal Encounters (FE) from 2000 to 2021 [5-8]. The only independent collection that is still active is Mapping Police Violence, which did not begin tracking PCH until 2013. FE has not updated its dataset since 2021, but it extends back the farthest to 2000. The validity of these independent data collections is still unknown including how complete these collections are and whether they are coding PCH to conform with the FBI definition. Researchers who want to study PCH before 2000 are left with the two official data sources with known data limitations. From 2000 to 2013, FE was an additional option, but it is unknown how complete and reliable this dataset is, especially in the 2000s before tracking and Google alerts were implemented.

In this ecological study, we attempt to correct for the missing data problem in SHR in order to conduct research on PCH before 2000. We use two different imputation techniques. The first is performed at the monthly level to obtain estimates of PCH across our 170-city sample from 1980 to 2010. We assess the validity of FE for 2001-2003 and 2011-2013 in a comparison to our imputed estimates. We notice a potential flaw in this data as the totals for 2001-2003 are lower compared to both imputed and non-imputed SHR totals and to later totals of FE in 2011-2013. It appears from our comparisons that FE estimates are not reliable before 2011. We also perform imputation on a three-year sum of PCH in order to perform a negative binomial random-effects analysis on PCH from 1980 to 2010 to illustrate whether results are dependent on imputation decisions. We analyze predictors based on prior studies of PCH [9-16], which includes minority threat theory, community violence, and social disorganization indicators. We find the killing of police officers and social disorganization to be significant predictors of PCH across both non-imputed and imputed specifications of PCH.

## Materials and methods

Our sample consists of the 170 U.S. cities that had populations greater than 100,000 in 1980. We assess PCH using SHR data in three-year sums from 1981-1983, 1991-1993, 2001-2003, and 2011-2013. We measure PCH one year after the decennial Census (1981, 1991, 2001, and 2011) to ensure that PCH will not overlap with independent variable measurement in our analysis. In our negative binomial random-effects analysis, all explanatory variables are measured in the Census years of 1980, 1990, 2000, and 2010. We do not collect data from NVSS for several reasons. First, SHR gives estimates on PCH that are 29% larger than NVSS estimates [1-3]. Second, missing data (SHR) can be corrected with imputation, but measurement error (NVSS) cannot be easily corrected. Third, there is greater comparability across studies with SHR as it is used in virtually all prior studies on police-caused homicides [9-16]. 

We impute SHR data to correct for missing data. SHR reports are submitted on a monthly basis by police departments, but this collection is treated as voluntary, as police agencies are not sanctioned for failing to submit a monthly report. In our sample of 12 years on 170 cities, SHR contained information for 16,727 months out of a possible 24,480 months (68% of total possible months) with reporting being higher in the 1980s and 1990s. This is mainly attributable to Florida, which stopped reporting SHR data in 1997. Multiple imputation that controlled for month, year, and city characteristics is employed for months with missing information. Twenty iterations of imputations are averaged to fill in values for unreported months in SHR in order to obtain total estimates of PCH for our 170-city sample.

As a validity check, we compare non-imputed and imputed SHR police-caused homicide totals to FE to evaluate how effectively imputed SHR totals adjust for missing data. We are able to compare SHR and FE totals for the last two analysis waves, 2001-2003 and 2011-2013. FE identified 2,873 police-caused homicides in these six years compared to 1,494 non-imputed SHR cases and 2,380 imputed SHR cases. FE data includes many cases that would not be categorized as a police-caused homicide in SHR, which are described as ‘felon killed by police’. The SHR definition includes a suspect who is killed after attacking a police officer or citizen, after fleeing or resisting arrest, and after or during committing a crime (1-3). Accidental deaths, suspects that commit suicide in front of officers, or suspects that kill innocent bystanders in front of police are not counted in the SHR definition, but they are counted in FE. Therefore, we code FE data to conform to the SHR definition of incidents where police intentionally kill a citizen. Each case in FE contains a description of the incident that allowed for this coding. After FE data were coded to conform to the SHR definition, the total number of PCH for FE was 2,043. This represents 71% of the total number of police-caused homicides listed in FE for our sample, which suggests that PCH will be overstated when using FE data without performing a coding analysis to conform to the FBI definition.

We impute SHR data for missing months in our sample in order to obtain estimates of PCH that correct for underreporting. These estimates are useful on their own to generate national estimates of PCH before 2000. Knowing how much PCH is underreported in official government statistics is imperative before any policy decisions can be made to address this issue. These estimates can also help evaluate the validity of independent data collections such as FE, which appears to be less accurate before 2010, as we describe later. Finally, these estimates helped identify errors in New York and Ohio that were easily spotted when comparing imputed estimates over time. New York and Ohio (except Toledo) are classified as missing data from 2011-2013, despite both of these states providing SHR reports to the FBI. Upon further investigation, we discovered that New York no longer specified police-caused homicides in their SHR reports starting in 2006. We also observed that no Ohio city in our sample, except Toledo, reported a police-caused homicide after 2010, which we know from FE comparisons is inaccurate. Therefore, we treat cities in New York and Ohio (except Toledo) as missing for our last analysis wave from 2011-2013, and we recommend that other researchers do the same.

For analysis purposes, there is usually little to gain from imputing the dependent variable [17]. This is also not the typical way to impute a dependent variable, which in this case will be a three-year sum of police-caused homicides. Multiple imputation would be conducted after these three-year sums are constructed for any remaining missing values. We perform this imputation technique as well and compare non-imputed and imputed results from negative binomial random-effects models. It is important to note that each three-year sum is based on 36 observations, or 36 months of data. As long as a city had at least one reported month of data, it would have a value for the three-year sum. Multiple imputation on the three-year sums rather than on each month would therefore not be able to correct for the number of reported months of data for cities that had at least one month of data. We specify that cities must have at least three months of data, or they are specified as missing. We also include results using our imputed estimates on the monthly data as a final comparison. The imputations performed at the monthly level that are averaged to obtain total estimates across cities are able to correct for the number of reported months across cities. This is an unorthodox imputation technique and is only included to demonstrate how robust results are to imputation techniques.

We do not claim that imputation on the three-year sums or on the months of missing data can completely overcome all SHR data limitations, but we do not believe these limitations can be completely ignored, as prior studies have done. The standard practice for all prior city-level studies that use SHR data on PCH is not to employ any imputation techniques and to use non-imputed multiple-year sums [9-16]. We do not agree with this approach as their estimates are not controlling for the number of reported months, which will affect their estimates of PCH. We recognize the limitations of this data and believe it is best to report alternative dependent variable specifications to address them. We conduct a negative binomial random-effects analysis on three-year sums of PCH with three different specifications: (i) No imputation and data treated as missing if they have less than three months of data, (ii) Imputation on the three-year sums for all cases with less than three months of data, and (iii) Imputation on the monthly data with 20 iterations averaged to compute three-year sums. We hope other researchers consider taking a similar approach when using SHR data to study police-caused homicides as a robustness check. 

Independent variables in our statistical analysis are measured in the census years of 1980, 1990, 2000, and 2010. We include predictors based on prior research [9-16] that include minority threat theory indicators that predict greater social control when racial/ethnic populations are high or increasing. We include percent Black, percent Hispanic, Black-White residential segregation, and Hispanic-White segregation. Segregation is measured using the index of dissimilarity that ranges from 0 to 100, with higher values representing greater segregation. We control for community violence levels that are predicted to be positively associated with PCH with two variables: violent crime rates (number of violent crimes known to police per 100,000) and a two-year sum of the number of police officers killed in a city. This measure is evaluated two years before the dependent variable is observed (1979-1980 and so on). Additional controls include police force size (police officers per 100,000 residents), an economic index, and a social disorganization index. The economic index is estimated using factor analysis on three economic variables: unemployment rate, median family income, and Gini index. The social disorganization index is computed using factor analysis on percent divorced, percent female-headed households, and percent of dwelling units with more than 1.01 residents per room (crowding). Year and regional dummies are included in all models. Some independent variables are logged to adjust for skewed distributional effects.

## Results

A descriptive analysis of SHR data for our 170-city sample reveals the extent of underreporting. Table 1 displays the number of months with reported data in SHR for each of the 12 years that are used to construct the three-year sums for police-caused homicide, with an average of 68% for reported months. In column 4 of Table 1, the total number of PCH in each year is presented. These are the raw amounts obtained from summing monthly totals. No imputation is conducted to correct for missing information.

**Table 1:** Police-Caused Homicide (PCH) Non-imputed and Imputed SHR Comparisons PCH: Police-Caused Homicide; SHR: Supplementary Homicide Reports Data Source: FBI Supplementary Homicide Reports

Year	Total Reported Months	Percentage of Reported Months	Total PCH Not Imputed	Total PCH Imputed	SHR Not Imputed/SHR Imputed
1981	1511	0.74	304	409	0.74
1982	1527	0.75	280	388	0.72
1983	1466	0.72	306	424	0.72
1991	1428	0.7	286	413	0.69
1992	1503	0.74	338	449	0.75
1993	1577	0.77	350	441	0.79
2001	1358	0.67	276	404	0.68
2002	1359	0.67	272	412	0.66
2003	1353	0.66	261	394	0.66
2011	1215	0.6	220	385	0.57
2012	1217	0.6	215	375	0.57
2013	1213	0.6	250	411	0.61
Total	16727	0.68	3358	4905	0.69

Table 1 column 5 presents the total number of PCHs when imputation is employed to correct for months with missing data. Twenty iterations of imputations are averaged to fill in values for unreported months in SHR in order to compute yearly totals. The last column in Table 1 shows what percentage the non-imputed SHR totals represent compared to the imputed SHR totals, which closely mirror the percentage of reported months. We find the SHR non-imputed estimates to be 32% lower compared to the imputed estimates.

As a validity check, we compare non-imputed and imputed SHR totals to FE to evaluate how effectively imputed SHR totals adjust for missing data. We are able to compare SHR and FE totals for the last two analysis waves, 2001-2003 and 2011-2013. Table 2 displays yearly comparisons for SHR and FE totals. SHR imputed totals are higher than FE totals for 2001-2003 and lower for 2011-2013. SHR non-imputed totals are also higher for 2001 and 2002 compared to FE totals. These comparisons indicate that FE totals are less reliable and likely underreported in the early 2000s compared to the 2010s.

**Table 2:** Police-Caused Homicide SHR and FE Comparisons PCH: Police-Caused Homicide; SHR: Supplementary Homicide Reports; FE: Fatal Encounters Data Source: FBI Supplementary Homicide Reports and Fatal Encounters

Year	Total PCH Not Imputed (SHR)	Total PCH Imputed (SHR)	Total PCH (FE)	SHR Not Imputed/FE	SHR Imputed/FE
2001	276	404	259	1.07	1.56
2002	272	412	250	1.09	1.65
2003	261	394	275	0.95	1.43
2011	220	385	407	0.54	0.95
2012	215	375	403	0.53	0.93
2013	250	411	449	0.56	0.92
Total	1494	2381	2043	0.73	1.17

Data collection for FE began in 2013 and these results may indicate that it is harder to collect PCH data the further back you go. One of the methods FE utilizes to collect PCH data is Google and newspaper article searches and it seems reasonable that this method would be less fruitful in the early 2000s, when newspapers had a lower online presence. Another method they use is tracking and Google alerts, which was not possible before the collection began in 2013. This is a limitation of FE data that hinders its validity before 2010. We recommend that researchers not use this data prior to 2010 without correcting for underreporting.

Focusing on 2011-2013, where FE data is more reliable, the non-imputed SHR totals are almost 50% lower compared to FE totals, as illustrated in column 5 of Table 2. This is a rather substantial disparity that hinders the validity of prior police-caused homicide research that utilizes SHR data without correcting for missing data. The imputed SHR totals are around 8% less from 2011-2013 compared to FE totals, which suggests multiple imputation techniques may be able to correct for most of the missing data in SHR, but not all. 

Finally, we conduct a statistical analysis of PCH using non-imputed and imputed measurements. Table 3 presents descriptive statistics and data sources for all analysis variables. Multiple imputation is employed to correct for missing data in explanatory variables, which consists of 22 out of the 680 observations (3%). All independent variables have been selected based on prior research on PCH, with studies typically controlling for racial/ethnic composition, crime/violence levels, economic indicators, and social disorganization (9-16). The purpose of our analysis is to illustrate how robust results are to different specifications of the dependent variable that include non-imputed and imputed estimates. 

**Table 3:** Descriptive Statistics U.S.: United States; FBI: Federal Bureau of Investigation; UCR: Uniform Crime Reports; NLEOMF: National Law Enforcement Officers Memorial Fund; SHR: Supplementary Homicide Reports

	Mean	Standard Deviation	Min	Max	N	Data Source
Independent Variables						
Percent Black	21.47	18.12	0.1	84.4	680	U.S. Census
Percent Hispanic	14.54	16.54	0.4	96.8	680	U.S. Census
Black-White Segregation	52.82	16.97	12.93	90.61	669	U.S. Census
Hispanic-White Segregation	38.01	12.98	9.02	70.71	669	U.S. Census
Violent Crime Rate	960.25	622.55	88.8	4352.8	672	FBI UCR
Killings of Police (2-year sum)	0.27	0.78	0	10	680	NLEOMF
Police Rate	222.44	88.97	85.35	805.57	679	FBI UCR
Economic Index	0	1	-3.28	3.92	680	U.S. Census
Social Disorganization Index	0	1	-2.55	4.7	680	U.S. Census
Dependent Variable						
Police-Caused Homicide (3-year sum)	5.46	11.38	0	106	623	FBI SHR

Table 4 presents negative binomial random-effects models on police-caused homicides. Negative binomial estimation is employed for all presented models because the dependent variable is a count variable, and alpha tests indicate that the data are over-dispersed, which indicates that a negative binomial estimation is preferable to a Poisson estimation [18]. City population is specified as an exposure variable in all models to account for the number of times the outcome could have happened [19]. The use of exposure variables is superior in many instances to analyzing rates as an explanatory variable because it corrects for differences in the probability distributions of the event across (in this case) cities [19]. Table 4 displays three specifications of police-caused homicides: non-imputed (model 1), imputed on three-year sum (model 2), and imputed on monthly data with values averaged to construct three-year sums (model 3).

**Table 4:** Negative Binomial Random-Effects Models on Police-Caused Homicide *Significant 5% level. **Significant 1% level. Standard errors in parenthesis. Note 1: All models include Year and Regional Dummies. Note 2: Each model uses different specifications of police-caused homicide: Model 1=non-imputed at least three months of data, Model 2: Imputed on 3-year sums, Model 3: Imputed on monthly data with values averaged to construct three-year sums.

Variables	Model 1	Model 2	Model 3
Percent Black	0.009*	0.006	0.005
	(0.005)	(0.005)	(0.004)
Percent Hispanic	-0.006	-0.005	-0.005
	(0.004)	(0.005)	(0.003)
Black-White Segregation (log)	0.161	0.091	-0.095
	(0.222)	(0.224)	(0.166)
Hispanic-White Segregation (log)	-0.032	-0.056	-0.257*
	(0.162)	(0.163)	(0.118)
Violent Crime Rate (log)	0.159	0.181	0.007
	(0.107)	(0.115)	(0.075)
Killings of Police	0.063*	0.097**	0.046**
	(0.025)	(0.027)	(0.017)
Police Rate (log)	0.127	0.089	0.091
	(0.176)	(0.186)	(0.143)
Economic Index	0.1	0.135	0.084
	(0.075)	(0.078)	(0.055)
Social Disorganization Index	0.273**	0.213*	0.263**
	(0.079)	(0.084)	(0.062)
Sample Size	623	680	680
Number of Cities	168	170	170

The results appear to be similar across the three different dependent variable specifications. There are two variables that are significant across all models: the killing of police officers and the social disorganization index. Both are positively signed, which is consistent with theoretical predictions. Percent Black is significant in model 1 that does not employ imputation techniques to correct for missing data in SHR. Hispanic-White segregation is significant in model 3 that is imputed on monthly data with values averaged to construct three-year sums. No other variables are significant in Table 4.

## Discussion

Our imputed estimates of police-caused homicides on a sample of 170 large U.S. cities are useful for analyzing trends from 1980 to 2010. According to the non-imputed three-year estimates of PCH using SHR data, this homicide outcome has decreased by 24% from 1981-1983 to 2011-2013. When adjusting for missing months of data using our imputed estimates, the decrease is observed to be only 4%. We also observe that the amount of missing data in SHR has increased from an average of 26% to 40% over this period. There is no data available for this homicide outcome after 2010 for the states of Florida and New York in SHR. Ohio also seems to no longer specify police-caused homicides in SHR with the exception of Toledo. Missing data has always been a limitation of SHR data and it appears that this problem has worsened over time. 

Our examination of FE data that extends back to 2000 reveals that this data appears to suffer from underreporting before 2010. We also discovered that 29% of FE incidents do not conform to the FBI definition of police-caused homicides for our sample of 170 large U.S. cities. We recommend that researchers who wish to use FE to study PCH not use this data prior to 2010 and to perform data coding to ensure that the amount of PCH is not overestimated.

For researchers who use SHR data to perform statistical analysis on police-caused homicides, we recommend reporting both non-imputed and imputed results. We present an example analysis in this study and find that two variables appear to be robust to imputation techniques. The killing of police officers is a positive and significant predictor across both non-imputed and imputed models. This indicates community violence effects are observed through direct effects to officer safety (killings of police) rather than indirect or adversary effects (violent crime rates). Killings of police may result in retaliatory policing strategies that lead to temporary increases in police use of force [20]. The findings of this study, coupled with other research on killings of police, indicate a potential reciprocal relationship between these two homicide outcomes [21,22]. This cycle of violence between the police and citizens may be hard to break in areas that have a high amount of both killings by and of police.

We also find social disorganization to be a positive predictor of police-caused homicides. Social disorganization has been positively linked to criminal homicides and it appears to also be linked to police-caused homicides [23]. Similar to community violence arguments, social disorganization may alter officers’ perceptions of residents in an area, making them more likely to define situations as requiring force, including lethal force [13]. It is encouraging that both significant findings in this analysis are consistent with theoretical predictions, but the purpose was to demonstrate whether findings would be robust to imputation techniques rather than to make any causal claims. 

Some scholars have argued that SHR data should not be used at all to study police-caused homicide [1], while others have justified its continued use [16]. We argue that SHR data problems should not be ignored and provide one potential solution in this paper by employing imputation techniques. We recognize the limitations of this data and try to address them as best as we can by presenting results for both non-imputed and imputed dependent variable specifications of police-caused homicides. 

## Conclusions

FBI SHR missing data limitations for police-caused homicides have increased since 1980. FE, an independent data collection of police-caused homicides, appears to suffer from underreporting before 2010. Researchers who wish to study police-caused homicides in an analysis that extends before 2010 will need to use SHR data. Given the missing data limitations in SHR, we recommend that researchers who use this data to study police-caused homicides report both non-imputed and imputed estimates in their results as a robustness check.
